# Chromosome passenger complex is required for the survival of cells with ring chromosomes in fission yeast

**DOI:** 10.1371/journal.pone.0190523

**Published:** 2018-01-03

**Authors:** Ahmed G. K. Habib, Kanako Sugiura, Masaru Ueno

**Affiliations:** 1 Department of Molecular Biotechnology, Graduate School of Advanced Sciences of Matter, Hiroshima University, Kagamiyama, Higashi-Hiroshima, Japan; 2 Department of Biotechnology and Life Sciences, Faculty of Postgraduate Studies for Advanced Sciences (PSAS), Beni-Suef University, Beni-Suef, Egypt; Tulane University Health Sciences Center, UNITED STATES

## Abstract

Ring chromosomes are circular chromosomal abnormalities that have been reported in association with some genetic disorders and cancers. In *Schizosaccharomyces pombe*, lack of function of protection of telomere 1 (Pot1) or telomerase catalytic subunit (Trt1) results in survivors with circular chromosomes. Hitherto, it is poorly understood how cells with circular chromosomes survive and how circular chromosomes are maintained. Fission yeast Cut17/Bir1, Ark1, Pic1, and Nbl1 is a conserved chromosome passenger complex (CPC) functioning mainly throughout mitosis. Here, using a temperature-sensitive mutant of CPC subunits, we determined that CPC is synthetically lethal in combination with either Pot1 or Trt1. The *pot1Δ pic1-T269* double mutant, which has circular chromosomes, showed a high percentage of chromosome mis-segregation and DNA damage foci at 33°C. We furthermore found that neither Shugoshin Sgo2 nor heterochromatin protein Swi6, which contribute to the centromeric localization of CPC, were required for the survival in the absence of Pot1. Both the *pot1Δ sgo2Δ* and *pot1Δ swi6Δ* double mutants displayed a high percentage of DNA damage foci, but a low percentage of chromosome mis-segregation, suggesting the link between the high percentage of chromosome mis-segregation and the lethality of the *CPC pot1Δ* double mutant. Our results suggest that CPC is required for the survival of cells with circular chromosomes and sheds light on the possible roles of CPC in the maintenance of circular chromosomes.

## Introduction

Ring chromosomes are circular DNA molecules that can be formed either by DNA-double strand breaks at both arms of a chromosome, which generates sticky ends ready for fusion [1–3], or by telomere dysfunction as a result of telomere uncapping [4–6]. Although ring chromosomes are rare in general, they evidently affect the cells in which they are present. Ring chromosomes are associated with various clinical phenotypes such as craniofacial dysmorphisms, intellectual disability, growth retardation, and epileptic seizures [7, 8]. Also, they have been reported in some cancers including malignant mesenchymal neoplasia (10%), dermatofibrosarcoma protuberans (70%), acute myelogenous leukemia, atypical lipomatous tumors (63%), acute lymphoblastic leukemia (3.4%), malignant mesenchymoma, parosteal osteosarcoma, and some others [9–16].

Telomeres, the DNA-protein structures at the termini of eukaryotic chromosomes, are important to maintain genomic integrity and protect chromosomes from end-to-end fusion, aberrant repair and degradation. Telomere dysfunction and degradation resulting from uncapping of a telomere trigger genomic instability, end-to-end fusion of chromosomes and is one of the causes for the formation of ring and dicentric chromosomes associated with some genetic diseases and cancers [17–19]. Some human tumors exhibit significantly shortened telomeric repeat sequences, which may initiate telomeric fusions between chromosome arms. Such fusions lead to formation of unstable ring and dicentric chromosomes that at cell division form bridges, which may break and result in novel chromosome rearrangements by fusion of the broken ends [20, 21].

The fission yeast *Schizosaccharomyces pombe* has been used for a long time as a model organism to study many cellular biological processes in eukaryotes due to the ease of genetic manipulation and the conservation of many eukaryotic genes in this strain. In fission yeast, cells compensate for telomere loss through chromosome circularization. For instance, lack of function of Pot1 or Trt1 results in telomere loss and survivors with circular chromosomes [22, 23]. Nevertheless, very little is known about how cells with circular chromosomes can survive. To better understand this, we used synthetic lethality approach. Synthetic lethality is a condition in which cell death occurs as a result of combining a mutation in a single gene with a mutation in another single gene [24]. Synthetic lethal interaction would exist between two genes in a redundant or similar essential pathway. In this study, we investigated a gene whose mutation results in death of cells with circular chromosomes.

In the few past decades, the chromosome passenger complex (CPC) has become one of the targets for anti-cancer drugs [25–27]. CPC is one of the highly conserved complexes orchestrating various events in cell division starting from chromosome condensation in prophase to cytokinesis [28, 29]. Accordingly, CPC is a crucial player in mitosis that ensures faithful chromosome segregation during cell division to avoid chromosome instability (CIN) and aneuploidy, which are the hallmarks of genetic diseases and cancers. CPC is a ternary complex consisting of the catalytic subunit Aurora kinase B, scaffolding subunit inner centromeric protein (INCENP), Survivin, and Borealin [30]. Homologs of this complex have been identified in fission yeast as Aurora B/Ark1, INCENP/Pic1, Survivin/Bir1, and Borealin/Nbl1 [31–33]. In fission yeast, the localization pattern of this complex spans the inner centromere during metaphase to spindle midzone in anaphase and midbody in telophase, and closely resembles their human counterpart [29, 34]. Knockdown or mutation of any of the CPC subunits results in highly comparable phenotypes such as chromosome congression and segregation defects in yeast, fly, worm, and mammalian cells [35–37]. Furthermore, the percentage of tetraploidy and cell death increases as a result of impaired cytokinesis.

Here, we have investigated the role of CPC in the survival of cells with ring chromosomes. We find that the lack of function of CPC was lethal to fission yeast *pot1Δ* and *trt1Δ* cells with circular chromosomes. Sgo2 and Swi6 were not synthetically lethal with Pot1, implying that the residual centromeric localization of CPC can sustain cell viability in the absence of Pot1. These findings demonstrate the importance of CPC in the survival of cells with ring chromosomes and suggest possible roles of CPC in the maintenance of circular chromosomes.

## Materials and methods

### Strain construction and growth media

The strains used in this study are listed in Table 1. The *pot1Δ cut17-275* (*pot1*::*kanMX6 cut17-275*), *pot1Δ bir1-T1* (*pot1*::*kanMX6 bir1-T1*), *pot1Δ pic1-T269* (*pot1*::*kanMX6 pic1-T269*), *pot1Δ ark1-T7* (*pot1*::*kanMX6 ark1-T7*), and *pot1Δ ark1-T8* (*pot1*::*kanMX6 ark1-T8*) double mutants expressing Pot1 from plasmid (pPC27- pot1^+^-hemagglutinin [HA], containing the *leu1* gene) were constructed by mating *h*^*−*^ *leu1 cut17-275*, *h90 ade6-M216 leu1 bir1-T1<<kanr*, *h*^*−*^ *leu1 pic1-T269<<hygR ade6-M216*, *h*^*−*^ *leu1 ade6 ark1-T7<<kanR Z*::*Padh 15 mCherry-atb2*^*+*^*<<natR* and *h90 ade6-M216 leu1 ark1-T8-GFP<<kanR*, respectively, with *h*^*+*^ *leu1-32 ura4-D18 ade6-M210 pot1*::*kanMX6* expressing Pot1 from the plasmid pPC27-leu1-pot1^+^-HA (AGK 004). Cells were streaked on Edinburgh minimal medium (EMM) lacking leucine to select candidates that retained the Pot1 plasmid and streaked on yeast extract agar (YEA) plates containing G418 disulfide at 25°C to select for the *pot1*::*kanMX6* mutation. Cells were re-streaked on YEA at 36°C to select the *cut17-275*, *bir1-T1*, *pic1-T269*, *ark1-T7*, and *ark1-T8* mutation, respectively. Cells that could grow at the permissive temperature (25°C) but not at the restrictive temperature (36°C) were selected as a double mutant.

**Table 1:** *Schizosaccharomyces pombe* strains used in this study.

Strain	Genotype	Source
FY9361	*h*^*−*^ *leu1 cut17-275*	NBRP
AGK004	*h*^*+*^ *leu1-32 ura4-D18 ade6-M210 pot1*::*kanMX6 (pPC27-Leu1-pot1*^*+*^*-HA)*	Lab freeze stock
AGK026	*h*^*−*^ *leu1 ade6-M210 pot1*:: *kanMX6 cut17-275 (pPC27- Leu1- pot1*^*+*^*-HA)*	This study
FY24496	*h*^*90*^ *ade6-M216 leu1 bir1-T1<<kanr*	NBRP
AGK027	*h*^*+*^ *leu1-32 ura4-D18 ade6-M210 pot1*:: *kanMX6 bir1-T1<<kanr (pPC27-Leu1- pot1*^*+*^*-HA)*	This study
FY24764	*h*^*−*^ *leu1 ade6-M216 pic1-T269<<hygR*	NBRP
SU001	*h*^*+*^ *leu1-32 ura4-D18 ade6-M216 pot1*::*kanMX6 pic1-T269<<hygR (pPC27-Leu1- pot1*^*+*^*-HA)*	This study
SU002	*h*^*+*^ *leu1-32 ura4-D18 ade6-M216 pot1*::*kanMX6 pic1-T269<<hygR*	This study
FY24593	*h*^*−*^ *leu1 ade6 ark1-T7<<kanR Z*::*Padh 15 mCherry-atb2*^*+*^*<<natR*	NBRP
SU005	*h*^*+*^ *leu1-32 ura4-D18 ade6 pot1*::*kanMX6 ark1-T7<<kanR Z*::*Padh 15 mCherry-atb2*^*+*^*<<natR (pPC27-Leu1- pot1*^*+*^*-HA)*	This study
FY24484	*h*^*90*^ *ade6-M216 leu1 ark1-T8-GFP<<kanR*	NBRP
SU006	*h*^*+*^ *leu1-32 ura4-D18 ade6 pot1*::*kanMX6 ark1-T8-GFP<<kanR (pPC27-Leu1- pot1*^*+*^*-HA)*	This study
NK310	*h*^*+*^ *leu1-32 ura4-D18 ade6-M210 trt1*::*kanMX6 pPC96-trt1*^*+*^	S. Ukimori
AGK120	*h*^*−*^ *leu1 ura4-D18 ade6-M216 trt1*::*kanMX6 pic1-T269<<hygR pPC96-trt1*^*+*^	This Study
AGK121	*h*^*−*^ *leu1 ura4-D18 ade6-M216 trt1*::*kanMX6 pic1-T269<<hygR*	This Study
FY13784	*h*^*90*^ *leu1 ade6-M210 ura4-D18 sgo2*::*ura4*^*+*^	NBRP
AGK009	*h*^*+*^ *leu1 ade6-M210 ura4-D18 pot1*::*kanMX6 sgo2*::*ura4*^*+*^ *(pPC27-Leu1-pot1*^*+*^*-HA)*	This study
AGK010	*h*^*+*^ *leu1 ade6-M210 ura4-D18 pot1*::*kanMX6 sgo2*::*ura4*^*+*^	This Study
FY13725	*h*^*90*^ *leu1 ade6-M210 ura4-D18 swi6*::*ura4*^*+*^	NBRP
AGK014	*h*^*+*^ *leu1 ade6-M210 ura4-D18 pot1*::*kanMX6 swi6*::*ura4*^*+*^ *(pPC27-Leu1-pot1*^*+*^*-HA)*	This study
AGK015	*h*^*+*^ *leu1 ade6-M210 ura4-D18 pot1*::*kanMX6 swi6*::*ura4*^*+*^	This Study
TN004	*h*^*+*^ *rad11-mRFP*::*natMX6*	T. Nanbu
KTA038	*h*^*−*^ *leu1-32 ura4-D18 ade6 pot1*::*kanMX6 rad11- mRFP*::*natMX6*	T. Nanbu
SU003	*h*^*−*^ *leu1 ade6-M216 pic1-T269<<hygR rad11-mRFP*::*natMX6*	This study
SU004	*h*^*+*^ *leu1-32 ura4-D18 ade6-M216 pot1*::*kanMX6 pic1-T269<<hygR rad11-mRFP*::*natMX6*	This study
AGK089	*h*^*90*^ *leu1 ade6-M210 ura4-D18 sgo2*::*ura4*^*+*^ *rad11-mRFP*::*natMX6*	This Study
AGK090	*h*^*90*^ *leu1 ade6-M210 ura4-D18 swi6*::*ura4*^*+*^ *rad11-mRFP*::*natMX6*	This Study
AGK091	*h*^*+*^ *leu1 ade6-M210 ura4-D18 pot1*::*kanMX6 sgo2*::*ura4*^*+*^ *rad11-mRFP*::*natMX6*	This Study
AGK092	*h*^*+*^ *leu1 ade6-M210 ura4-D18 pot1*::*kanMX6 swi6*::*ura4*^*+*^ *rad11-mRFP*::*natMX6*	This Study

The *trt1Δ pic1-T269* (*trt1*::*kanMX6 pic1-T269*) double mutants were constructed by mating *h*^*+*^ *trt1*::*kanMX6* cells (NK310) expressing Trt1 from plasmid (pPC96-*trt1*^*+*^, a gift from Professor Toru Nakamura, containing *ade6*^*+*^ and *HSV-tk*^+^ as a positive and negative selection marker, respectively) with *h*^*−*^ *pic1-T269* (FY24764). Cells were streaked on EMM lacking adenine to select candidates that retained the Trt1 plasmid and on YEA plates containing G418 disulfide at 25°C to select for *trt1*::*kanMX6* mutation. Cells that could grow at 25°C but not at 36°C were selected as a double mutant. YEA plates containing 100 μM 2-deoxy-5-fluorouridine (FUDR) were used as a counter-selection medium for cells that lost the Pot1 or Trt1 plasmid.

*pot1Δ sgo2Δ* (*pot1*::*kanMX6 sgo2*::*ura4*) and *pot1Δ swi6Δ* (*pot1*::*kanMX6 swi6*::*ura4*) double mutants harboring the Pot1 plasmid (pPC27- pot1^+^- hemagglutinin [HA], containing the *leu1* gene) were constructed by mating *h*^*+*^ *pot1*::*kanMX6* expressing Pot1 from the plasmid pPC27-leu1-pot1^+^-HA (AGK 004) with *h90 sgo2*:: *ura4*^*+*^ *leu1 ade6-M210 ura4-D18* and *h90 swi6*::*ura4*^*+*^ *ura4-D18 leu1 ade6-M210*, respectively. Candidates were streaked on EMM plus adenine (EMM+A) to select cells that retained the Pot1 plasmid and *sgo2*::*ura4* (in the case of *pot1Δ sgo2Δ*) and the Pot1 plasmid and *swi6*::*ura4* (in the case of *pot1Δ swi6Δ*). Cells were re-streaked on YEA plates containing G418 disulfide at 30°C to select candidates with the *pot1*:*kanMX6* mutation.

To tag the Rad11 protein with monomeric red fluorescent protein (mRFP) at the C-terminus, pFA6a-mRFP-natMX6-rad11 was linearized by NspV and used in the transformation of FY24764 SU002, FY13784, FY13725, AGK010, and AGK015 strains, resulting in SU003, SU004, AGK089, AGK090, AGK091, and AGK092 strains, respectively. Cells were grown in YEA medium (0.5% yeast extract, 3% glucose, and 40 μg/ml adenine) or EMM with the required supplements at the indicated temperatures.

### Measurement of telomere length

Telomere length was measured using Southern hybridization as previously described protocol [38] with an AlkPhos Direct Kit (GE Healthcare). For probing, the telomere-associated sequence plus telomere fragment digested with EcoRI derived from pNSU70 was used.

### Pulsed-field gel electrophoresis (PFGE)

PFGE was performed as previously described [39]. For the detection of NotI-digested chromosomes, *S*. *pombe* NotI-digested chromosomal DNA was fractionated in a 1% agarose gel with 0.5 × TBE (50 mM Tris–HCl, 5 mM boric acid, and 1 mM EDTA [pH 8.0]) buffer using the CHEF Mapper PFGE system at 6 V/cm (200 V) and a pulse time of 60–120 s for 24 h. DNA was visualized by staining with ethidium bromide (1 μg/ml) for 30 min.

### Microscopy

Microscope images of living cells were obtained using an AxioCam digital camera (Zeiss) connected to an Axio Observer Z1 microscope (Zeiss) with a plan-Apochromat 63 × objective lens (numerical aperture, 1.4). Pictures were captured and analyzed using AxioVision Rel. 4.8.2 software (Zeiss).

### Lactose gradient synchronization

One hundred milliliters of cell culture were grown in YEA to mid-log phase (5×10^6^ cells/ml) at 25°C. Ten milliliters of 20% lactose solution was prepared in a 15 ml Falcon tube, frozen at -80°C for 4 h, and then thawed without disturbance for 3 h at 30°C to generate a 10–30% gradient. Cells were harvested by centrifugation at 3000 rpm for 3 min and the cell pellet was re-suspended in 750 μl of sterile water. The cell suspension was layered on top of the lactose gradient using cut-off blue tips and centrifuged at 1000 rpm for 8 min, during which the cells formed a smear about half-way down the gradient. Fractions of about 0.1–0.4 ml were quickly removed from just below the top of the smear using cut-off blue tips. The cells were harvested by centrifugation at 4000 rpm for 30 sec in an Eppendorf tube, re-suspended in YEA medium, and examined under the microscope for the uniformly small early G2 cells.

## Results

### Pot1 is synthetically lethal with CPC components

**Fig 1:**
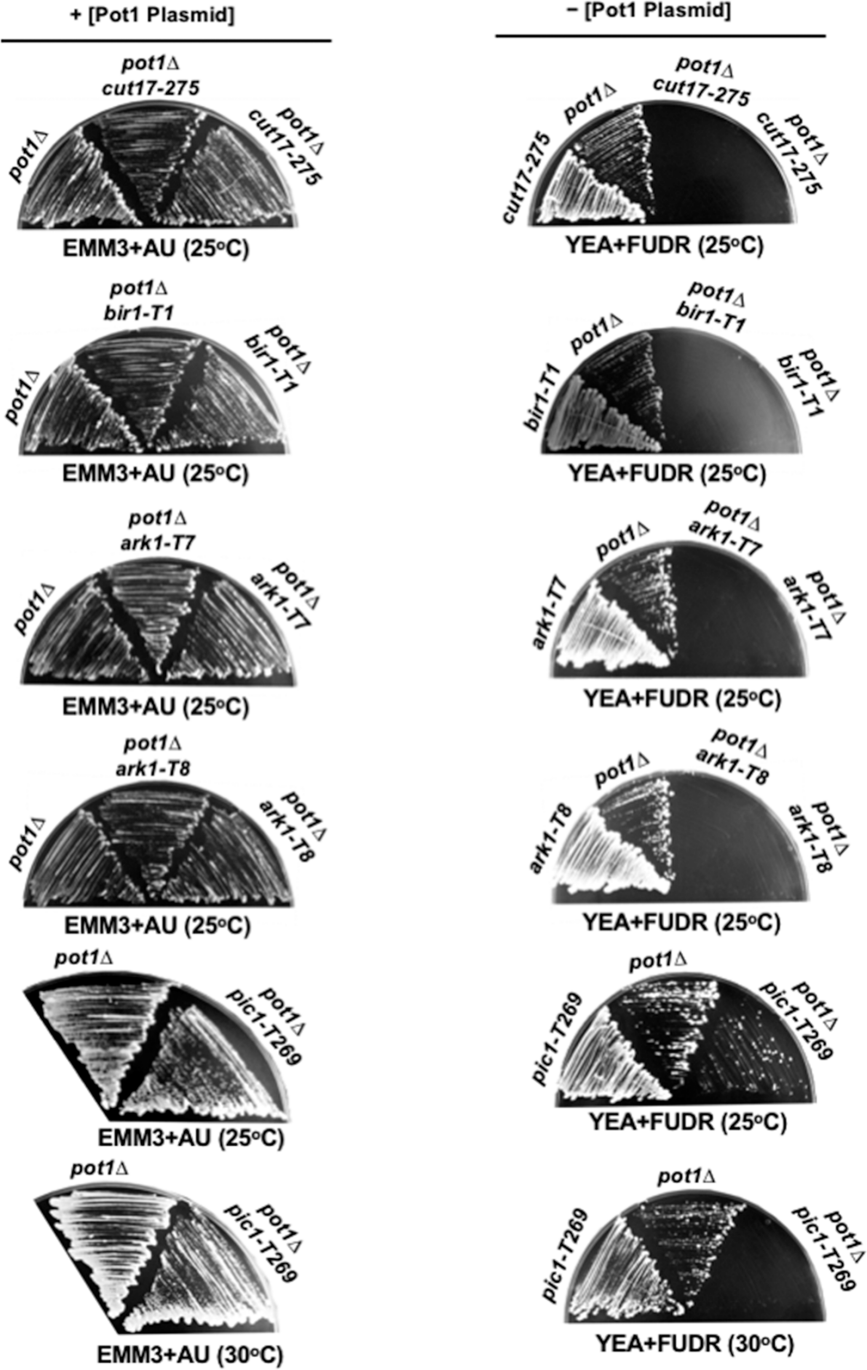
Survival of the double mutants before and after loss of Pot1 plasmid. The *pot1Δ cut17-275*, *pot1Δ bir1-T1*, *pot1Δ ark1-T7*, *pot1Δ ark1-T8* and *pot1Δ pic1-T269* double mutants carrying plasmid-borne *pot1*^*+*^ and *tk*^*+*^ were streaked on selective and counter-selective media at the indicated temperatures. Pot1 plasmid was retained on EMM plates with adenine and uracil (EMM+AU). FUDR-containing plates were used as a counter selection to examine the ability of cells to grow after loss of the Pot1 plasmid.

Since *pot1Δ* cells can survive only through chromosome circularization, we used the *pot1Δ* strain to identify the gene that is involved in the maintenance of the circular chromosome. To investigate whether CPC is required for the survival of cells with circular chromosomes, we constructed double mutants between Pot1 and CPC subunits (Cut17/Bir1, Ark1, and Pic1) and examined the ability of the double mutants to survive. Knowing that CPC is essential for cell viability, we used temperature-sensitive mutant alleles of CPC subunits as follows: *cut17-275* (A990T), *bir1-T1*, which displays a better growth phenotype than *cut17-275* at the permissive temperature [40], *ark1-T7*, *ark1-T8*, and *pic1-T269*. The permissive temperature of these temperature-sensitive mutants was 25°C. The restrictive temperature for *cut17-275*, *bir1-T1*, and *pic1-T269* was 36°C, and for *ark1-T7* and *ark1-T8* was 33°C (S1 Fig). The *pot1Δ cut17-275*, *pot1Δ bir1-T1*, *pot1Δ pic1-T269*, *pot1Δ ark1-T7* and *pot1Δ ark1-T8* constructed strains carry a plasmid containing *pot1*^*+*^ in addition to the gene for thymidine kinase (*tk*^*+*^), which is used as a negative selection marker. The expression of *tk*^*+*^ in the presence of FUDR is lethal to cells. Therefore, FUDR-containing plates were used as to counter-select cells able to grow after the loss of the plasmid. We found that all double mutants retaining the Pot1 plasmid could grow. However, the *pot1Δ cut17-275*, *pot1Δ bir1-T1*, *pot1Δ ark1-T7*, and *pot1Δ ark1-T8* double mutants failed to grow after the loss of the Pot1 plasmid even at 25°C (Fig 1). In the case of the *pot1Δ pic1-T269* double mutant, some colonies could grow at 25°C after the loss of Pot1 plasmid, however, the cells lost the viability at 30°C (Fig 1). These results indicate that Pot1 is synthetically lethal with CPC and point out the importance of CPC for the survival of cells with ring chromosomes.

### *pot1Δ pic1-T269* double mutant survivors have lost telomeric DNA and harbor circular chromosomes

**Fig 2:**
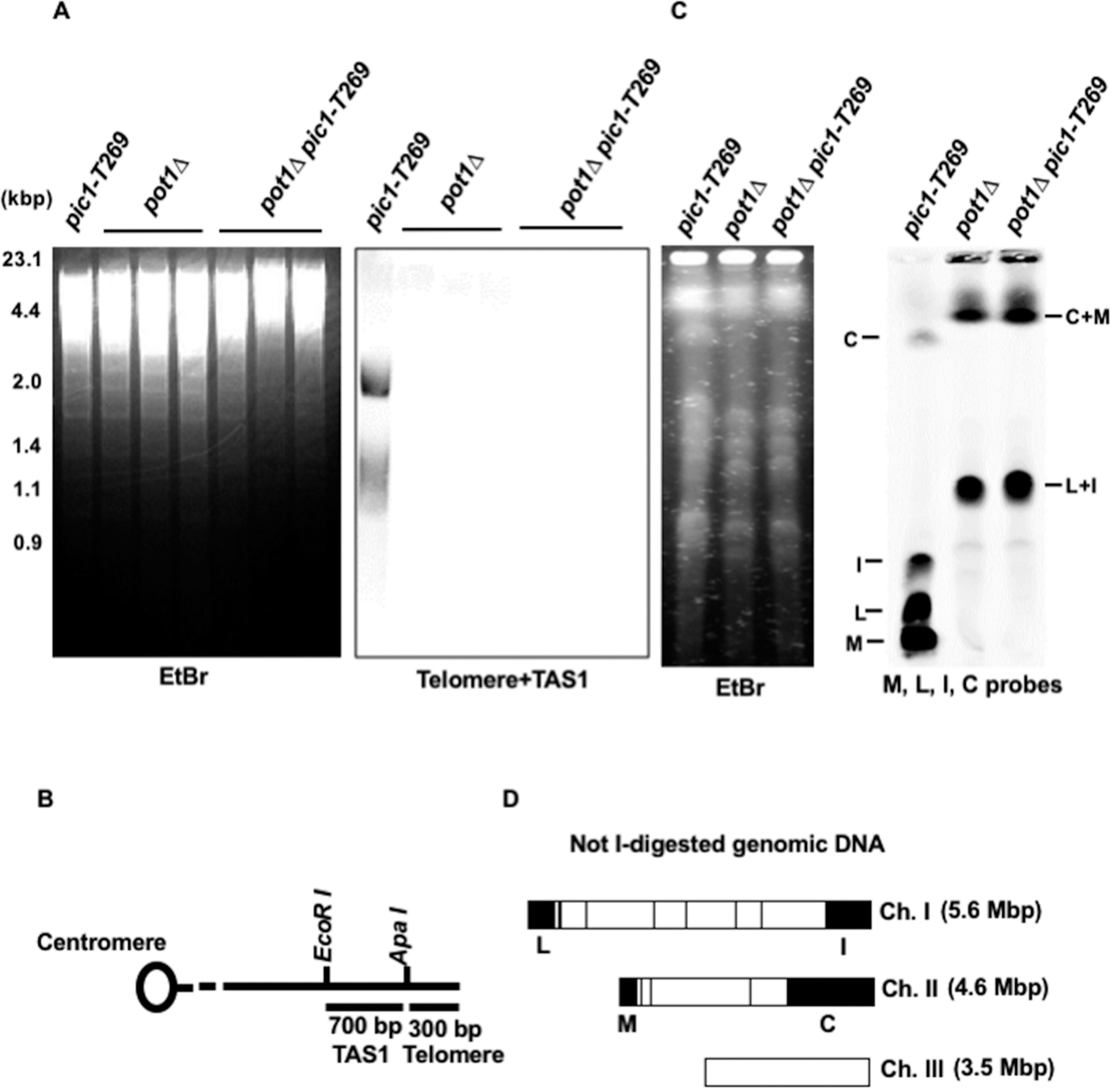
*pot1Δ pic1-T269* double mutants lose telomeric DNA and have circular chromosomes. (A) The telomere length of *pot1Δ pic1-T269* double mutants was analyzed by Southern hybridization at 25°C. *pot1Δ* and *pic1-T269* single mutants were used as a control for strains that lost and retained the telomeric DNA, respectively. Genomic DNA was digested by EcoRI and fractionated by 1.5% agarose gel electrophoresis. Telomere plus telomere associated sequence (TSA1) derived from pNSU70 was used as a probe for hybridization. To assess the total amount of DNA, the gel was stained with ethidium bromide (EtBr) before blotting onto the membrane. (B) Restriction enzyme sites around the telomere and TAS1 of one chromosome arm cloned in the plasmid pNSU70. (C) NotI-digested chromosomal DNA from *pic1-T269*, *pot1Δ* and *pot1Δ pic1-T269* cells were analyzed by PFGE at 25°C. The digested DNA fractionated in a 1% agarose gel and a mixture of four probes (L, I, C, M) specific to the NotI-digested chromosomal terminal fragments were used. (D) NotI restriction enzyme map of *S*. *pombe* chromosomes.

To determine whether the *pot1Δ pic1-T269* double mutant that survives at 25°C maintains the circular chromosome phenotype, genomic DNA from the *pot1Δ pic1-T269* double mutant was analyzed by Southern blotting at 25°C. DNA was digested with EcoRI and the telomeric repeats were examined utilizing a probe containing telomere and telomere-associated sequence 1 (TAS1). *pot1Δ* and *pic1-T269* single mutants were used as control strains for cells with circular and linear chromosomes, respectively. We found that the *pot1Δ pic1-T269* double mutant completely lost the telomeric hybridization signal, similar to the *pot1Δ* single mutant (Fig 2A and 2B). To further confirm that the *pot1Δ pic1-T269* double mutant lacked linear chromosomes and harbored circular chromosomes, the genomic DNA was digested with NotI and analyzed by PFGE at 25°C. We found that the NotI-digested fragments M, L, I, and C, which are located at the end of chromosome I and II, were lost and bands corresponding to C+M and L+I were detected (Fig 2C and 2D). These results mirrored the behavior of cells having circular chromosomes and confirm that the *pot1Δ pic1-T269* survivors have circular chromosomes.

### Pic1 is required for the viability of *trt1Δ* cells having circular chromosomes

**Fig 3:**
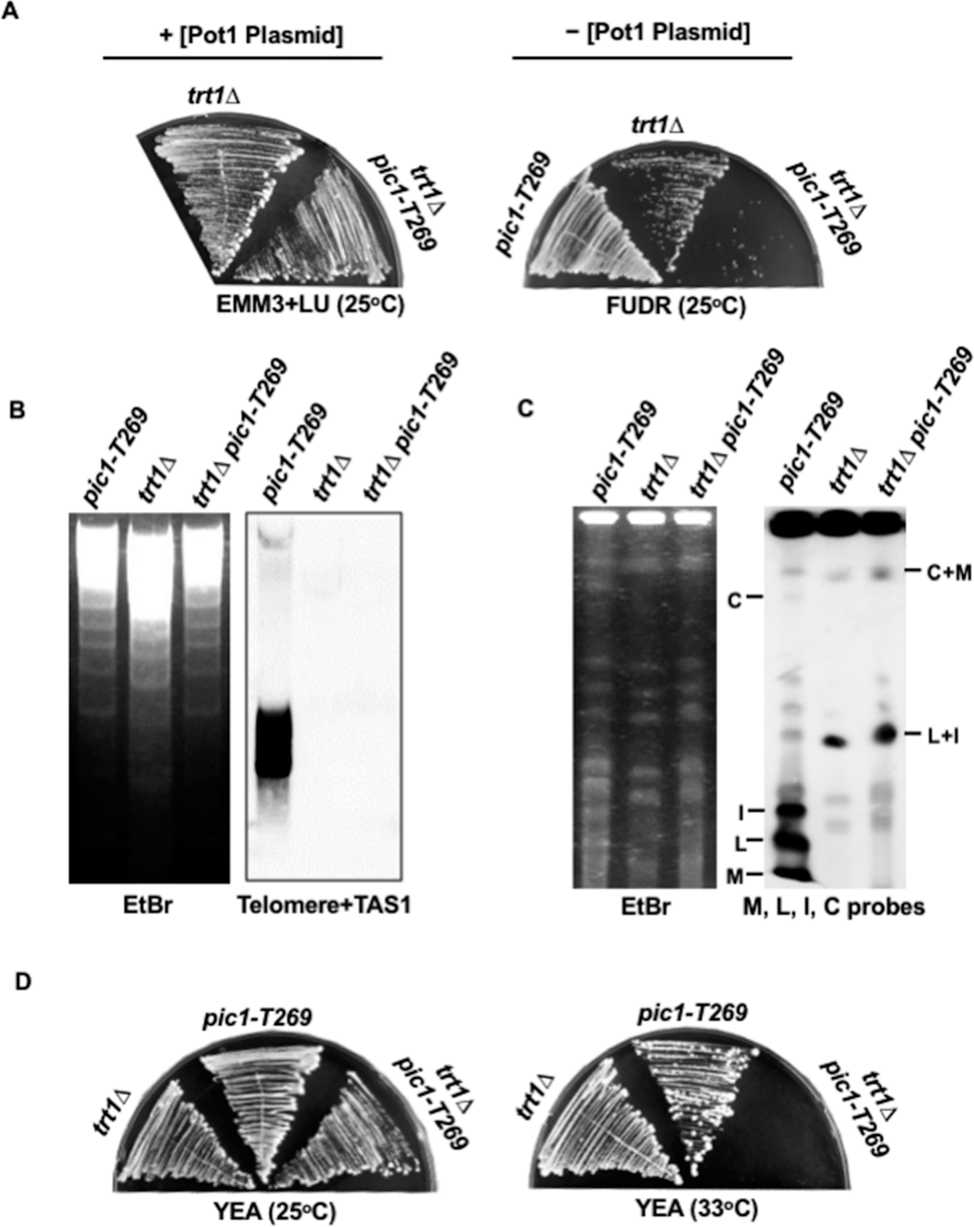
Pic1 is required for the survival of *trt1Δ* cells having circular chromosomes. (A) *trt1Δ pic1-T269* cells were streaked on YEA+FUDR plates to select for cells that could grow after the loss of plasmid expressing *trt1*^*+*^ and *tk*^*+*^. The plasmid was retained on EMM plates supplemented with leucine and uracil (EMM+LU). (B) *trt1Δ pic1-T269* double mutants lost telomeric DNA. The loss of telomeric DNA in *trt1Δ pic1-T269* double mutant survivors was analyzed by Southern hybridization at 25°C. (C) NotI-digested chromosomal DNA from *pic1-T269*, *trt1Δ* and *trt1Δ pic1-T269* cells were analyzed by PFGE at 25°C. (D) Lack of function of Pic1 results in loss of the viability of *trt1Δ* with circular chromosome. *trt1Δ pic1-T269* double mutant cells having circular chromosomes were streaked on YEA plates at 33°C to examine the ability of the cells to grow. *trt1Δ* with circular chromosomes and *pic1-T269* were used as controls.

Our observation that Pot1 is synthetically lethal with CPC raised the question of whether this lethality is peculiar to Pot1 or is a generic phenotype for other cells with circular chromosomes. To address this question, we investigated the synthetic lethality between CPC and another mutant that displays the circular chromosome phenotype. In fission yeast, deletion of *trt1*^*+*^ encoding the catalytic subunit of telomerase results in gradual attrition of the telomere and progressive loss of viability, producing cell progeny with circular chromosomes [23]. Given that only the *pot1Δ pic1-T269* double mutant was able to survive at 25°C, we examined the synthetic lethality between Trt1 and Pic1. We constructed the *trt1Δ pic1-T269* double mutant that harbors a plasmid expressing *trt1*^*+*^ and *tk*^*+*^, and examined the ability of the cells to grow after the loss of the plasmid using FUDR-containing plates. We found that the *trt1Δ pic1-T269* double mutants produced colonies at 25°C (Fig 3A). We next examined the loss of telomeric DNA by Southern blotting and chromosome circularization by PFGE in *trt1Δ pic1-T269* cells, as described for the *pot1Δ pic1-T269* double mutant (see Fig 2). We found that some of *trt1Δ pic1-T269* double mutant cells completely lost telomeric DNA and harbored circular chromosomes (Fig 3B and 3C). Using these cells, we examined the ability of the *trt1Δ pic1-T269* double mutant having circular chromosomes to grow on YEA at the semi-permissive temperature of 33°C, the temperature at which Pic1 partially loses its function. We found that the *trt1Δ pic1-T269* double mutant completely lost the ability to grow at 33°C (Fig 3D), indicating that the *trt1Δ pic1-T269* double mutant is also synthetically lethal, and implying that functional Pic1 is required to sustain the viability of *trt1Δ* cells having circular chromosomes. This result affirms the importance of CPC for the survival of cells with circular chromosomes and supports the notion that the genetic interaction between CPC and *pot1*^*+*^ is not specific, but it is a prevailing phenotype of cells with circular chromosomes.

### *pot1Δ pic1-T269* double mutant loses viability with time and displays elevated rates of chromosome segregation defects and DNA damage foci at 33°C

**Fig 4:**
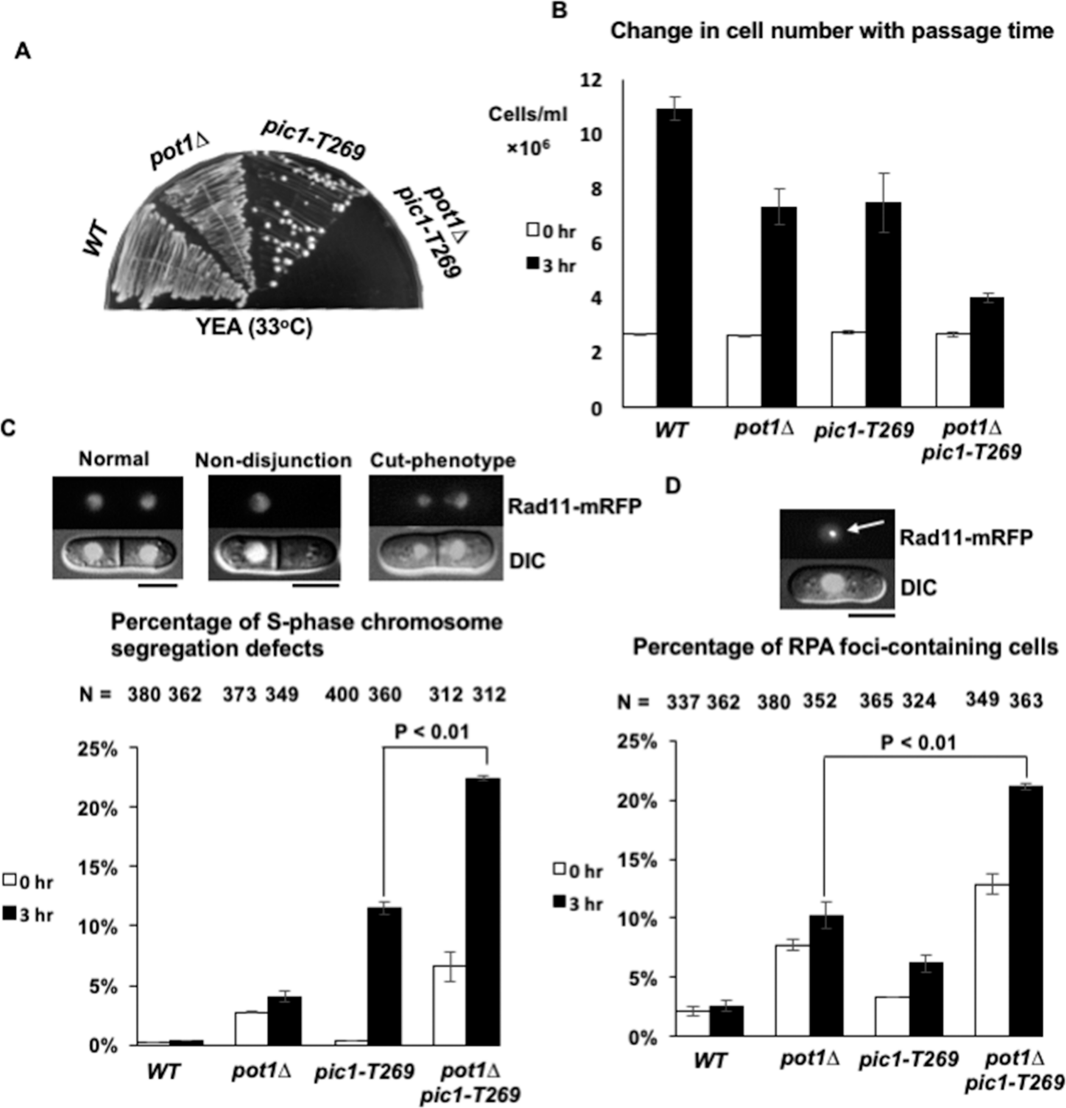
Analysis of the synthetic lethality phenotypes associated with the lack of function of Pic1 in *pot1Δ* cells. (A) *pot1Δ pic1-T269* double mutant cells lose viability at semi-permissive temperature (33°C). *Wild-type* (*WT*), *pot1Δ*, *pic1-T269*, and *pot1Δ pic1-T269* cells were streaked on YEA at 33°C. (B) Change in the cell number with time at 33°C. *WT*, *pot1Δ*, *pic1-T269*, and *pot1Δ pic1-T269* cells were incubated overnight at 25°C. An equal cell density (2.62×10^6^ cells/ml) of each strain was shifted to 33°C for 3 h. The change in the cell number after 3 h was calculated using a hemocytometer and compared. Error bars represent standard deviation (SD) from three independent experiments (n = 3). (C) Calculation of the percentage of chromosome segregation defects with septum in asynchronous living cells. *WT*, *pot1Δ*, *pic1-T269*, and *pot1Δ pic1-T269* living cells expressing Rad11 endogenously tagged with mRFP were incubated overnight at 25°C and shifted to 33°C for 3 h. The percentage of chromosome segregation defects at 25°C and 33°C was scored and compared. Representative images of cells that have chromosome segregation defects such as cut phenotype and chromosome non-disjunction are shown. (D) The percentage of RFP foci-containing cells was calculated at 25°C and after the 3-h shift at 33°C using the data from chromosome segregation defects analysis. The arrow indicates RPA foci. N in the top refers to the number of cells examined. Error bars represent SD (n = 3 experiments). The scale bar represents 5 μm.

As the *pot1Δ pic1-T269* double mutant failed to grow on YEA at 33°C, while the *pic1-T269* single mutant grew (Fig 4A), we measured the change in cell number of the double mutant with the passage of time after a temperature shift to 33°C in liquid culture. An advantage of monitoring growth in liquid culture is the ability to quantitatively detect subtle changes in the growth profile of the cells. By increasing the temperature from 25°C to 33°C, Pic1p in *pic1-T269* cells partially loses its function. Therefore, if Pic1 is important for the survival of cells with circular chromosomes, a subtle decrease in its function after temperature shift would result in loss of the viability of *pot1Δ* cells. To test this, wild type (*WT*), *pot1Δ*, *pic1-T269* and *pot1Δ pic1-T269* strains were cultured overnight at 25°C and then the cells were shifted to 33°C for 3 h. The change in the cell number after temperature shift was determined and compared. We found that the *pot1Δ pic1-T269* double mutant experienced notably growth defect compared to *pot1Δ* and *pic1-T269* single mutants after temperature shift (Fig 4B), indicating that functional CPC is important for the survival of cells with circular chromosomes.

The existence of *pot1Δ pic1-T269* double mutant survivors that could grow at 25°C prompted us to investigate the phenotypes associated with the depletion of CPC subunits, in this case Pic1, in *pot1Δ* cells. Since chromosome mis-segregation is a remarkable phenotype associated with CPC dysfunction, one possible phenotype to be examined is the accumulation of chromosome mis-segregation events. Moreover, some studies linked chromosome segregation errors and the occurrence of DNA damage [41, 42]. Therefore, we investigated whether the synthetic lethality of the *pot1Δ pic1-T269* double mutant is associated with elevated rates of chromosome segregation defects and DNA damage. To this end, Rad11, which encodes for the large subunit of replication protein A (RPA), was tagged with monomeric red fluorescent protein (mRFP) and used as a marker for chromosome segregation and DNA damage foci simultaneously. RPA is a known marker of single-stranded DNA that accumulates during DNA replication, damage, and repair processes. Examples of the chromosome mis-segregation events we examined are cut-phenotype, uncoupling of nuclear and cellular division resulting in septum tearing segregated chromosomes, and chromosome non-disjunction. *WT*, *pot1Δ*, *pic1-T269*, and *pot1Δ pic1-T269* cells expressing Rad11-mRFP were incubated overnight at 25°C then shifted to 33°C for 3 h. The percentage of chromosome segregation defects and DNA damage foci were scored at both 25°C and 33°C. We observed an increase in both chromosome segregation defects and DNA damage foci in *pot1Δ pic1-T269* double mutants compared to *pot1Δ* and *pic1-T269* single mutants even at 25°C (Fig 4C and 4D). These results imply that the elevated rates of chromosome segregation defects and the accumulation of DNA damage may be the cause of the synthetic lethality phenotype of the *pot1Δ pic1-T269* double mutant.

### Formation of RPA foci in *pot1Δ pic1-T269* does not directly link to chromosome mis-segregation events

**Fig 5:**
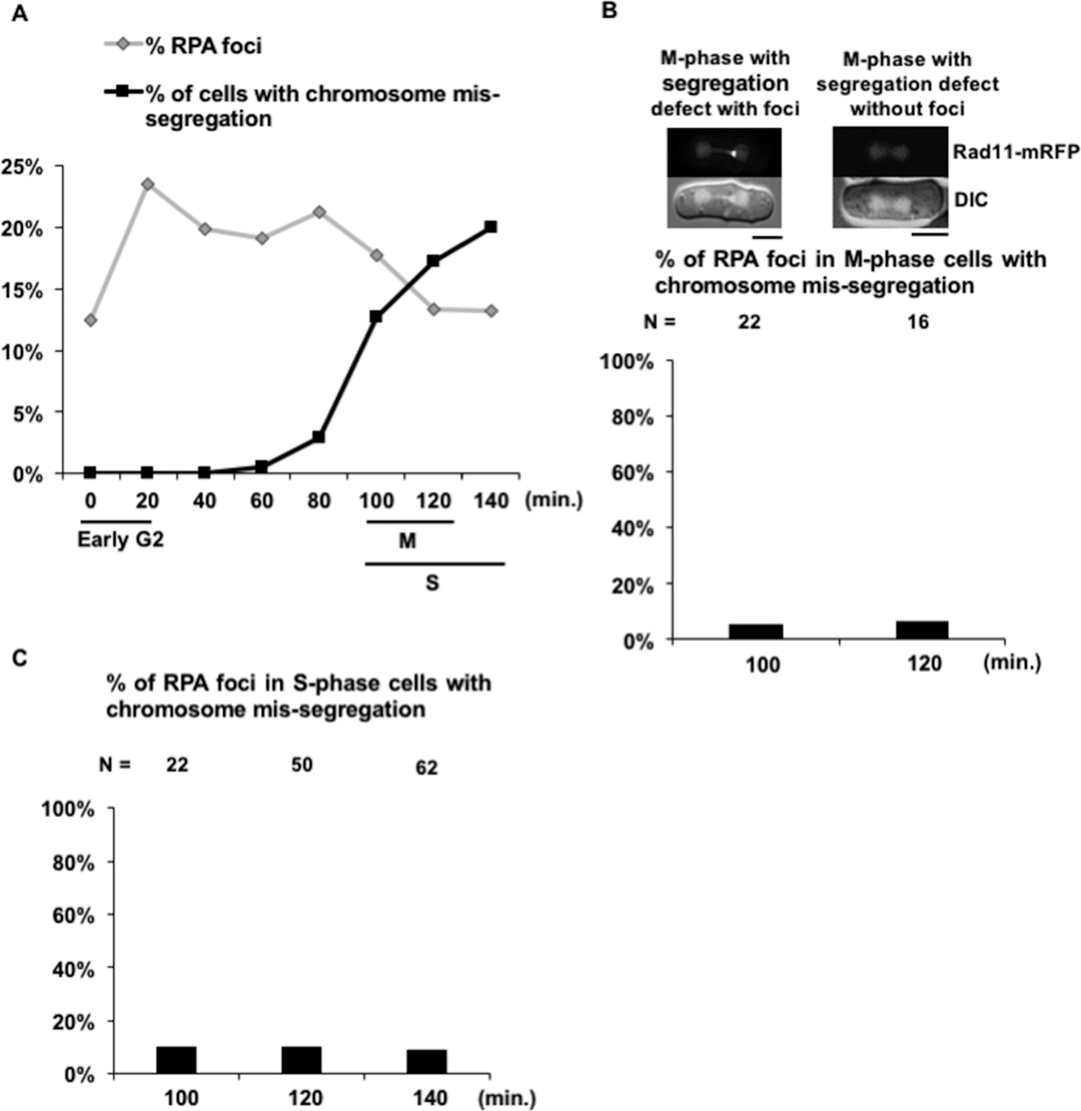
Correlation between DNA damage and chromosome mis-segregation in the *pot1Δ pic1-T269* double mutant. (A) The percentage of RPA foci and chromosome mis-segregation in *pot1Δ pic1-T269* synchronized cells. *pot1Δ pic1-T269* cells harboring Rad11 endogenously tagged with mRFP were incubated overnight at 25°C. Then, the cells were synchronized by lactose gradient centrifugation producing cells at early G2, shifted to 33°C with sampling every 20 min, and scored for the percentage of RPA foci and chromosome mis-segregation. The time point (0) corresponds to the overnight culture at 25°C before temperature shift. M cells, septated cells (as a marker for S phase), and early G2 cells are shown by bars (see also S2 Fig). (B) The percentage of RPA foci in mitotic cells with chromosome mis-segregation 100 and 120 min after temperature shift to 33°C. The scale bar represents 5 μm. Examples of cells and the total number of cells observed (N) in this experiment are shown on the top. (C) The percentage of RPA foci in septating S-phase cells with chromosome mis-segregation 100, 120, and 140 min after temperature shift to 33°C.

The high percentage of RPA foci and chromosome mis-segregation patterns observed in the *pot1Δ pic1-T269* double mutant prompted us to ask whether there is a link between the chromosome mis-segregation and the accumulation of DNA damage foci. To investigate this, we monitored the percentage of RPA foci and chromosome segregation defects at each stage of the cell cycle by utilizing the lactose gradient synchronization method that synchronizes the cells at early G2, marked with mono-nucleated small size cells. During the synchronization steps, *pot1Δ pic1-T269* cells were cultured at 25°C. Then, the synchronized cells were shifted to 33°C and sampled every 20 min. We found that the RPA foci were detected in G2 cells and the percentage of RPA foci did not increase at the time points corresponding to an increase in the percentage of M and S-phase cells with chromosome mis-segregation (Fig 5A). This result suggests that chromosome mis-segregation does not directly induce RPA foci in S-phase. Moreover, we scored the percentage of mitotic cells with chromosome mis-segregation displaying RPA foci. We found that a very small fraction (~5%) of cells with chromosome mis-segregation displayed RPA foci; in other words, the majority of cells with chromosome mis-segregation had no evidence of DNA damage (Fig 5B). This result ruled out the possibility that DNA damage in G2 phase induces chromosome mis-segregation. Next, to assess the possibility that the execution of cytokinesis on the mis-segregated chromosomes produces RPA foci, we scored the percentage of septated (S-phase) cells with chromosome mis-segregation displaying RPA foci. We found that a very small proportion (~10%) of septated cells with chromosome mis-segregation had RPA foci (Fig 5C), suggesting that cytokinesis does not induce RPA foci in S-phase, where the DNA damage response is active. Taken together, our results suggest that the formation of RPA foci in *pot1Δ pic1-T269* cells is not directly linked to the chromosome mis-segregation events.

### Loss of function of Shugoshin (Sgo2) or heterochromatin protein (Swi6) is not synthetically lethal with Pot1

**Fig 6:**
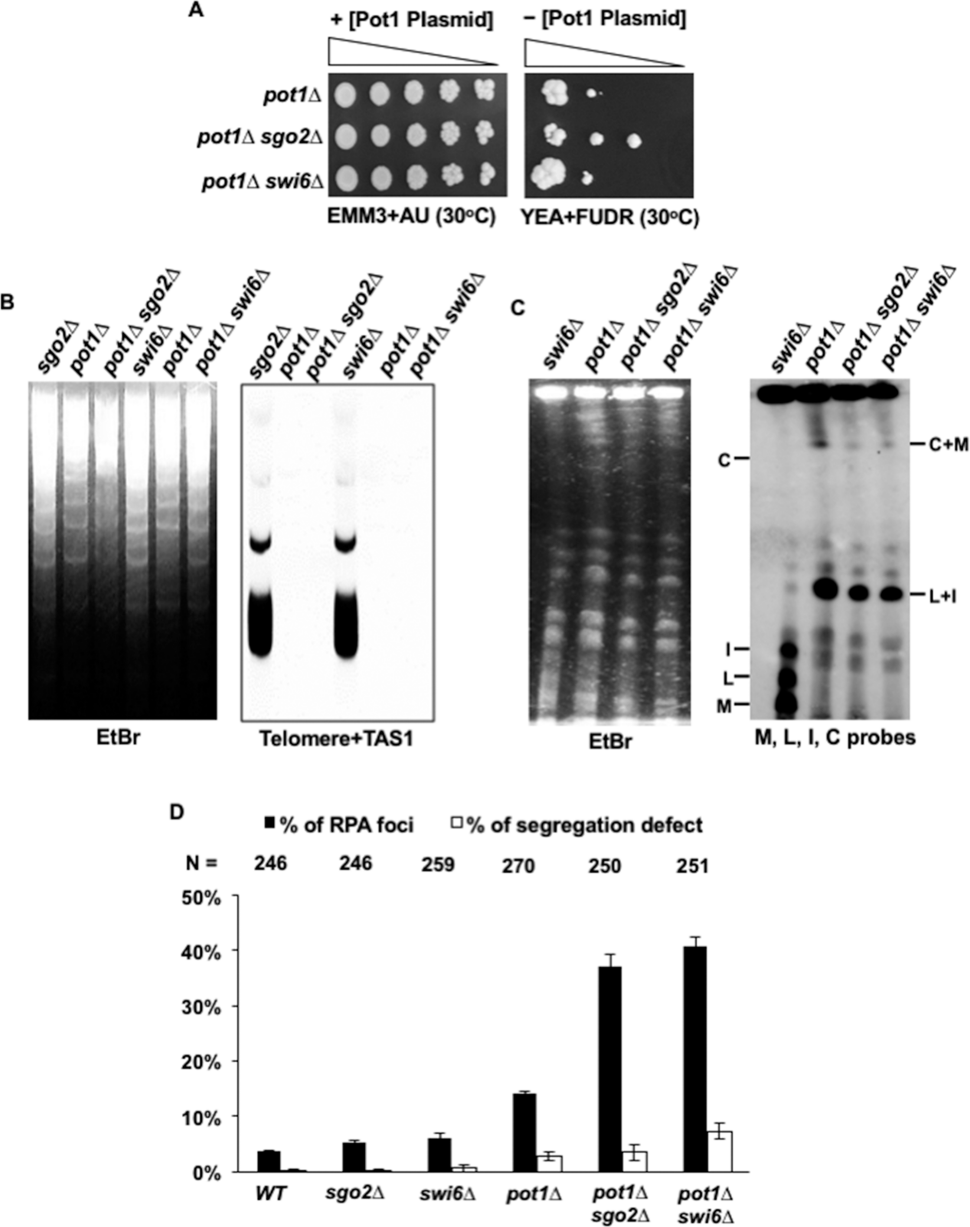
Sgo2 and Swi6 are not required for the survival of cells with circular chromosomes. (A) Spot assay of ten-fold serial dilutions of cells. *pot1Δ*, *pot1Δ sgo2Δ* and *pot1Δ swi6Δ* cells expressing Pot1 from plasmid were spotted on EMM+AU and YEA+FUDR plates at 30°C. The plasmid was retained on EMM+AU plates and cells that could grow after the loss of the plasmid were counter selected on YEA+FUDR at 30°C. (B) The telomere length of the *pot1Δ sgo2Δ* and *pot1Δ swi6Δ* double mutants was analyzed by Southern hybridization at 30°C. Both *sgo2Δ* and *swi6Δ* were used as a control for strains that retain telomeric DNA and *pot1Δ* cells as a control for strain that lost telomeric DNA. (C) NotI-digested chromosomal DNA from *swi6Δ*, *pot1Δ*, *pot1Δ sgo2Δ*, and *pot1Δ swi6Δ* cells were analyzed by PFGE at 30°C. (D) The percentage of RPA foci and chromosome mis-segregation in asynchronous living cells. The percentage of RPA foci and chromosome mis-segregation in *WT*, *sgo2Δ*, *swi6Δ*, *pot1Δ*, *pot1Δ sgo2Δ*, and *pot1Δ swi6Δ* cells harboring Rad11 endogenously-tagged with mRFP were simultaneously scored at 30°C. The total number of cells observed (N) in this experiment are shown on the top.

The fission yeast *S*. *pombe* has two members of the Shugoshin family, Sgo1 and Sgo2. While Sgo1 has only meiotic functions, Sgo2 has meiotic and mitotic roles [43]. It was previously reported that the deletion of *sgo2*^*+*^ results in a remarkable reduction in the centromeric localization of the Aurora kinase complex [40, 44]. In a like manner, the fission yeast heterochromatin protein Swi6 plays an important role in the centromeric localization of the Aurora kinase complex, and deletion of *swi6*^*+*^ results in a reduction in the centromeric localization of Aurora kinase complex [40]. This prompted the question of whether the deletion of either *swi6*^*+*^ or *sgo2*^*+*^ might exhibit a synthetic lethal interaction with *pot1Δ*. To test this possibility, we constructed *pot1Δ sgo2Δ* and *pot1Δ swi6Δ* double mutants carrying a plasmid containing *pot1*^*+*^ and *tk*^*+*^, and examined the ability of cells to grow after the loss of the Pot1 plasmid on FUDR-containing plates using a spot assay. We found that both double mutants were able to grow after the loss of the Pot1 plasmid. The colony formation efficiency of both *pot1Δ sgo2Δ* and *pot1Δ swi6Δ* cells was almost comparable to that of *pot1Δ* cells (Fig 6A), suggesting that deletion of *sgo2*^*+*^ or *swi6*^*+*^ does not influence the survival of *pot1* disruptant. We further examined the loss of telomeric DNA and chromosome circularization in *pot1Δ sgo2Δ* and *pot1Δ swi6Δ* cells using Southern blotting and PFGE as described for *pot1Δ pic1-T269* cells (see Fig 2). We found that both the *pot1Δ sgo2Δ* and *pot1Δ swi6Δ* double mutants completely lost the telomeric hybridization signal and that the chromosomes were circularized (Fig 6B and 6C). These results indicate that the *pot1Δ sgo2Δ* and *pot1Δ swi6Δ* double mutants are not synthetically lethal and imply that the residual accumulation of CPC is sufficient for the survival of cells with circular chromosomes.

### Percentage of RPA foci, but not aberrant chromosome segregation, increases in *pot1Δ sgo2Δ* and *pot1Δ swi6Δ* double mutants

To determine the reason behind the lack of synthetic lethality in the *pot1Δ sgo2Δ* and *pot1Δ swi6Δ* double mutants, we explored the phenotype of these double mutants in greater detail. If the high percentage of chromosome segregation defects and the RPA foci are the reasons for the synthetic lethality phenotype observed in the *pot1Δ pic1-T269* double mutant at 33°C, then we might expect that both the *pot1Δ sgo2Δ* and *pot1Δ swi6Δ* double mutants would display lower levels of chromosome segregation defects and RPA foci. To investigate this, we analyzed the percentage of chromosome segregation defects and the RPA foci using the p*ot1Δ sgo2Δ* and *pot1Δ swi6Δ* double mutants harboring Rad11 endogenously tagged with mRFP. Both the *pot1Δ sgo2Δ* and *pot1Δ swi6Δ* double mutants displayed a high percentage of RPA foci, but both showed a low percentage of chromosome mis-segregation compared to the *pot1Δ pic1-T269* double mutant (Fig 6D). These results suggest that the low percentage of aberrant chromosome segregation would be the reason for the lack of synthetic lethality phenotype observed in the *pot1Δ sgo2Δ and pot1Δ swi6Δ* double mutants.

## Discussion

Some studies reported the association of ring chromosomes with clinical disorders such as epilepsy, mental and developmental defects, as well as cancers [7–16]. However, little is known regarding the maintenance of circular chromosomes and how cells with circular chromosomes can survive. The unpredicted abnormalities that associate with the ring chromosome formation may stem from the unstable mitosis resulting from abnormal chromosome segregation and sister chromatid exchange [45]. In this study, we characterized the importance of CPC in the survival of cells with ring chromosomes using a synthetic lethality approach.

Throughout this study, we mainly used *pot1* disruptant as a model for a strain with circular chromosomes, since *pot1Δ* has the advantage of producing cells that can survive only through chromosome circularization [22]. Although chromosome circularization in fission yeast has been reported in other mutants [23, 46], some of these mutants can alternatively give rise to survivors with linear chromosomes [23].

To examine the importance of CPC for the survival of cells with circular chromosomes, we first generated a double mutant between *pot1Δ* and a temperature-sensitive mutant allele of *cut17/bir1*, and tested the ability of the *pot1Δ cut17-275* and *pot1Δ bir1-T1* double mutants to grow after the loss of plasmid-borne Pot1. We found that the double mutants lost the ability to grow after the loss of the Pot1 plasmid even at 25°C (Fig 1), indicating that the *pot1Δ cut17-275* and *pot1Δ bir1-T1* double mutants are synthetically lethal. Cut17/Survivin is detected in a complex with Pic1/INCENP and Ark1/Aurora B in many organisms. This complex is interdependent as the disruption of any of the CPC subunits leads to similar phenotypes [35, 47]. In budding yeast, the lack of function of the INCENP homolog (Sli15) and Aurora homolog (Ipl1) has identical phenotypes [37]. These findings are consistent with our observations that the other CPC subunits, Ark1 and Pic1, were also synthetically lethal with Pot1 (Fig 1). We further showed that the lack of function of Pic1 also resulted in death of *trt1Δ* cells having circular chromosomes (Fig 2D), indicating that CPC is required for survival of cells that have circular chromosomes and that it is not a specific genetic interaction with Pot1.

We further found that the functional inactivation of Pic1 by a temperature shift in *pot1Δ* cells, which have circular chromosomes, resulted in growth defects, accumulation of high rates of chromosome segregation defects, and increase in the percentage of DNA damage foci (Fig 4B, 4C and 4D). These results raise the possibility of a link between the chromosome segregation events and the formation of DNA damage foci. One possibility is that the RPA foci are produced earlier and induce chromosome mis-segregation events. It has been reported that the formation of pre-mitotic DNA damage that persists into mitosis can lead to chromosomal instability and segregation errors [48, 49]. However, our data suggest that this possibility is unlikely as we found that the majority of mitotic cells with chromosome mis-segregation had no sign of DNA damage (Fig 5B). Some reports linked aberrant chromosome segregation and the formation of DNA damage, suggesting that the entrapment of mis-segregated chromosome at the cleavage furrow during cytokinesis leads to chromosome breakage and generation of DNA damage [41, 42]. Therefore, a second possibility is that the DNA damage foci are produced after chromosome mis-segregation when the septum tears the mis-segregated chromosomes. If this is the case, then we would expect to observe a high percentage of septated S-phase cells with chromosome mis-segregation that display RPA foci. Instead, we found that the majority of the S-phase cells with chromosome mis-segregation that had septa did not have RPA foci (Fig 5C), indicating that this possibility is less likely. Nonetheless, it remains possible that the DNA damage foci are produced in the next G2 as a result of genomic instability arising from chromosome segregation errors. A third possibility is that the RPA foci formation and chromosome mis-segregation events are not directly linked. Indeed, we support this possibility since the first and second possibilities discussed above are less likely. Moreover, as will be discussed below, both the *pot1Δ sgo2Δ* and *pot1Δ swi6Δ* double mutants displayed a high percentage of DNA damage foci even though they had low rates of chromosome segregation defects, supporting the idea that DNA damage foci and chromosome segregation defects are not directly linked. In line with this, some studies showed no direct relationship between chromosome mis-segregation and the generation of DNA damage. [50, 51]. However, this possibility requires further investigation since there is no direct evidence yet.

We further investigated whether the reduction of the centromeric localization of CPC upon deletion of *sgo2*^*+*^ or *swi6*^*+*^ is lethal to cells with circular chromosomes. In humans, the dual inhibition of hSgo1 and Sgo2 by RNA interference results in reduction in the centromeric localization of the Aurora kinase complex [52]. Similar results have been observed in fission yeast upon deletion of either *sgo2*^*+*^ or *swi6*^*+*^ [40, 44]. Interestingly, we found that both the *pot1Δ sgo2Δ* and *pot1Δ swi6Δ* double mutants are viable, suggesting that the residual centromeric localization of CPC may be sufficient to sustain the viability of cells with circular chromosomes (Fig 6A). Consistent with this, both the *sgo2Δ* and *swi6Δ* single mutants were viable but deletion of CPC subunits was lethal. Our data also agrees with the finding that Swi6 is dispensable for the telomerase-minus *trt1Δ* circular survivors [53].

It has been shown that the lack of function of CPC subunits leads to higher rate of chromosome mis-segregation events than either *sgo2Δ* or *swi6Δ* single mutants. For instance, deletion of *sgo2*^*+*^ produces a mild effect on chromosome segregation in unperturbed cycling cells [44]. Even though both the *pot1Δ sgo2Δ* and *pot1Δ swi6Δ* double mutants displayed a high percentage of DNA damage foci, both consistently displayed a remarkably low percentage of chromosome mis-segregation compared to the *pot1Δ pic1-T269* double mutant (Fig 6D), suggesting that the elevated rates of chromosome mis-segregation per se, but not the DNA damage, is the likely reason for the synthetic lethality observed in *pot1Δ pic1-T269* cells at 33°C. This result further supports our suggestion that the link between chromosome mis-segregation and the generation of DNA damage foci in the *pot1Δ pic1-T269* double mutant is less likely. Our results also could raise a question regarding the possible roles of CPC, Sgo2, and Swi6 in the prevention of DNA damage when the chromosome is circular, which would be interesting to investigate.

## Supporting information

S1 Fig Cell growth of the temperature-sensitive mutant alleles of chromosome passenger complex (*cut17-275*, *bir1-T1*, *pic1-T269*, *ark1-T7* and *ark1-T8*) on YEA at the indicated temperatures. Note that both *ark1-T7* and *ark1-T8* cannot form colonies at 33°C, the temperature at which *cut17-275*, *bir1-T1*, and *pic1-T269* can still grow.
(TIF)

S2 Fig Cell cycle progression of the *pot1Δ pic1-T269* double mutant. Synchronous early G2 cells accumulated using lactose gradient centrifugation and progression through the cell cycle was monitored by sampling the cells every 20 min after temperature shift to 33°C. The percentages of M phase (two nuclei without septum) and S phase (two nuclei with septum) cells are shown.
(TIF)
