# Supplementary figures and images for: Chromosome passenger complex is required for the survival of cells with ring chromosomes in fission yeast
Source: PLoS One. 2018 Jan 3;13(1):e0190523. doi: 10.1371/journal.pone.0190523 (PMC5752009; doi:10.1371/journal.pone.0190523)

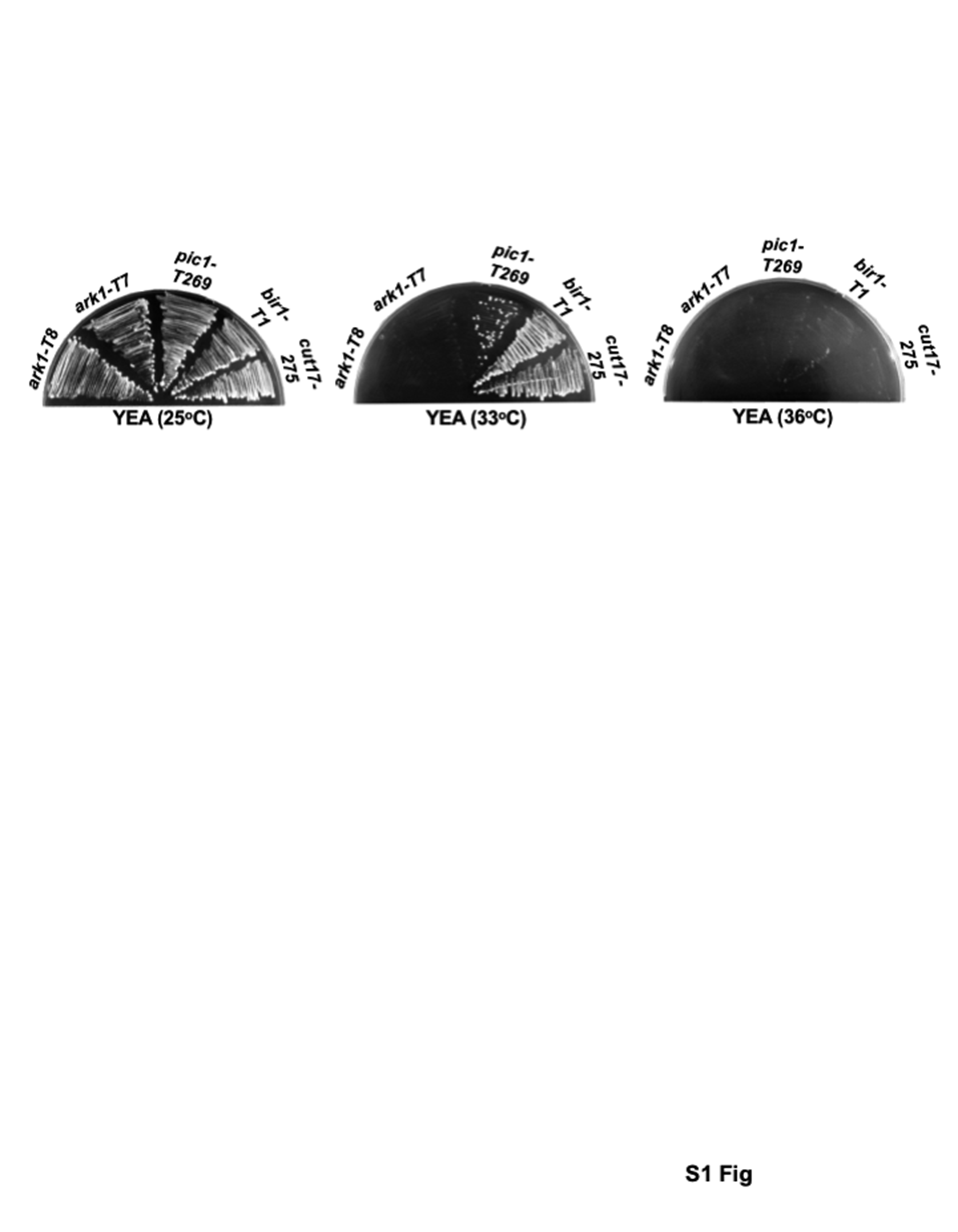

Supplement: S1 Fig — Note that both ark1-T7 and ark1-T8 cannot form colonies at 33°C, the temperature at which cut17-275, bir1-T1, and pic1-T269 can still grow. (TIF) [file pone.0190523.s001.tif]

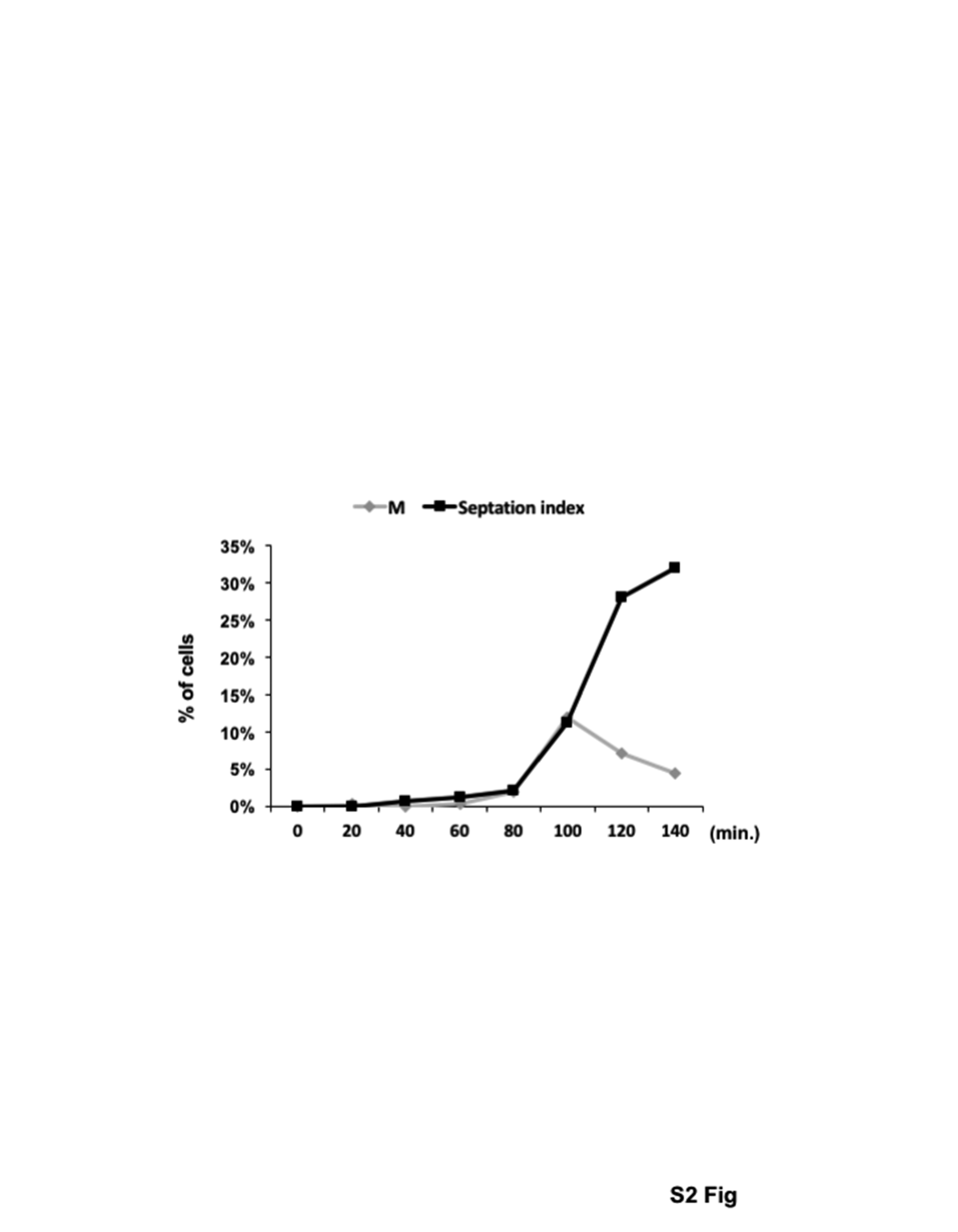

Supplement: S2 Fig — The percentages of M phase (two nuclei without septum) and S phase (two nuclei with septum) cells are shown. (TIF) [file pone.0190523.s002.tif]
